# Heat Shock Protein B1-Deficient Mice Display Impaired Wound Healing

**DOI:** 10.1371/journal.pone.0077383

**Published:** 2013-10-15

**Authors:** Jonathan Crowe, Anna Aubareda, Kay McNamee, Paulina M. Przybycien, Xin Lu, Richard O. Williams, George Bou-Gharios, Jeremy Saklatvala, Jonathan L. E. Dean

**Affiliations:** 1 Kennedy Institute of Rheumatology, Nuffield Department of Orthopaedics, Rheumatology and Musculoskeletal Sciences, University of Oxford, Oxford, United Kingdom; 2 Department of Medicine, Imperial College London, London, United Kingdom; 3 The Ludwig Institute for Cancer Research, Nuffield Department of Clinical Medicine, University of Oxford, Oxford, United Kingdom; University of São Paulo, Brazil

## Abstract

There is large literature describing *in vitro* experiments on heat shock protein (hsp)B1 but understanding of its function *in vivo* is limited to studies in mice overexpressing human hspB1 protein. Experiments in cells have shown that hspB1 has chaperone activity, a cytoprotective role, regulates inflammatory gene expression, and drives cell proliferation. To investigate the function of the protein *in vivo* we generated hspB1-deficient mice. HspB1-deficient fibroblasts display increased expression of the pro-inflammatory cytokine, interleukin-6, compared to wild-type cells, but reduced proliferation. HspB1-deficient fibroblasts exhibit reduced entry into S phase and increased expression of cyclin-dependent kinase inhibitors p27^kip1^ and p21^waf1^. The expression of hspB1 protein and mRNA is also controlled by the cell cycle. To investigate the physiological function of hspB1 in regulating inflammation and cell proliferation we used an excisional cutaneous wound healing model. There was a significant impairment in the rate of healing of wounds in hspB1-deficient mice, characterised by reduced re-epithelialisation and collagen deposition but also increased inflammation. HspB1 deficiency augments neutrophil infiltration in wounds, driven by increased chemokine (C-X-C motif) ligand 1 expression. This appears to be a general mechanism as similar results were obtained in the air-pouch and peritonitis models of acute inflammation.

## Introduction

The small heat shock protein (hsp)B1 (human hsp27 and its murine orthologue hsp25) is a member of the small hsp family that comprises 10 proteins, including the lens proteins αA- and αB-crystallin, and they all share a conserved C-terminal α-crystallin domain [1]. There is now a very extensive literature on the small heat shock protein, mainly describing functions proposed on the basis of experiments performed *in vitro*, or on cultured cells. HspB1 has been detected in a wide range of human cell types including fibroblasts [2], endothelial cells [3], macrophages [4] and neutrophils [5]. In contrast, little is known about its function *in vivo*, and the only murine strain reported to date with impaired hspB1 expression, a knockin mutant expressing a truncated form of hspB1 fused to a β-galactosidase reporter gene, does not appear to display any obvious phenotype [6].

HspB1 is an ATP-independent chaperone and regulates ubiquitin-mediated protein triage [7]. It is cytoprotective against hyperthermia [8] and a range of cytotoxic agents, including TNF [9], Fas/APO-1, staurosporine [10], H_2_O_2_ [11] and anticancer drugs [12]. Overexpression of human hspB1 in mice protects against apoptosis in kainite-induced seizures [13] and ischemia-reperfusion injury [14], [15], [16]. Various different mechanisms for the anti-apoptotic function of hspB1 have been proposed [17]. HspB1 is overexpressed in many different cancers [18] and recently has been suggested to promote cell proliferation [19], [20].

HspB1 was shown to be phosphorylated in human fibroblasts in response to IL-1 treatment some 20 years ago [21]. The activities responsible for phosphorylation of hspB1 were followed and were found to function in a kinase cascade, known as the p38 MAPK pathway [22], [23]. The pathway comprises the upstream activator, TGF-β-activated protein kinase [24], MAPK kinases-3 and -6 [25], [26], p38 MAPK itself, MAPK-activated protein kinase-2 [22], [23], and various downstream substrates including the RNA-binding protein, tristetraprolin [27], [28] and hspB1 [22], [23]. The p38 MAPK pathway is recognised to be one of the major pathways regulating inflammatory gene expression, exerting effects on both the transcription and stability of inflammatory mediator mRNAs [29]. Despite being an abundant substrate for the pathway, the function of the small heat shock protein in inflammation has remained obscure.

We previously found that depletion of hspB1 in HeLa cells inhibits the activation by IL-1 or TNF of pro-inflammatory signalling and induction of inflammatory response genes [2]. Inhibition of upstream signalling in HeLa cells [30] and inflammatory gene expression in human umbilical vein endothelial cells [31] and in an angiogenic breast cancer cell line [20] has been confirmed by others. However, depletion of the small heat shock protein by different siRNAs was found to increase TNF-induced nuclear factor-κB activation in HeLa [32] and TNF-induced prostaglandin E_2_ production and IL-8 expression in human keratinocytes [33]. The possibility of siRNA-associated off-target and cell type-dependent effects renders the function of hspB1 in inflammatory gene expression unclear and emphasises the need for a complete gene knockout.

To investigate the function of hspB1 *in vivo* we generated a new mouse strain in which all three exons of the hspB1 gene were flanked with LoxP sites and deleted using CRE-recombinase. As reported previously, a strain expressing a truncated form of *hspB1* fused to a β-galactosidase reporter gene [6], *hspB1* ^del/del^ mice are fertile and do not show any obvious abnormalities or spontaneous disease. Characterisation of hspB1-deficient fibroblasts showed that IL-1-induced IL-6 expression is increased and cell proliferation is reduced. Entry into S phase is inhibited and expression of the cyclin-dependent kinase inhibitor proteins, p21^waf1^ and p27^kip1^ is increased. In keeping with effects on the cell cycle, we also find that the expression of hspB1 is cell cycle-dependent. The defect in proliferation prompted us to investigate the function of hspB1 in cutaneous wound healing. We show that *hspB1* ^del/del^ mice display a significant impairment of wound healing due to not only a defect in proliferation but also exacerbated inflammation.

## Materials and Methods

### Mice

Targeting vector construction and knock-out strategy was designed and performed by genOway (Lyon, France). The genomic region of interest containing the murine *hspB1* locus was isolated by PCR from 129Sv/Pas ES cell genomic DNA. PCR fragments were subcloned into the pCRXL-TOPO or pCR4-TOPO vector (Invitrogen, Carlsbad, California). The resulting sequenced clones (containing whole *hspB1* gene sequence from promoter region to sequence downstream of exon 3) were used to construct the targeting vector. Briefly, a 3.3 kb region comprising exons 1 to 3 was flanked by a Neo cassette (*FRT* site-PGK promoter-Neo cDNA-FRT site-*Lox*P site) and a distal *Lox*P site in order to allow the generation of constitutive or conditional knock-out lines by deleting the whole gene (exons 1 to 3) of the *hspB1* gene. Linearised targeting vector was transfected into 129SvPas ES cells (genOway, Lyon, France) according to genOway’s electroporation procedures (ie 10^8^ ES cells in presence of 100 µg of linearised plasmid, 260 V, 500 µF). Positive selection was started 48 h after electroporation, by addition of 200 µg/ml of G418 (150 µg/ml of active component, Life Technologies, Inc.). 111 resistant clones were isolated and amplified in 96-well plates. The set of plates containing ES cell clones amplified on gelatin were genotyped by PCR : by amplification of the targeted locus: sense (Neo cassette), 5′-TGA CTA GGG GAG GAG TAG AAG GTG GC-3′); antisense 5′-TCT TGC TAC AAG CCT GGG ACT CTG G-3′). Recombination of the targeted locus was confirmed by Southern blot analysis using internal and external probes on both 3′ and 5′ ends. Two clones were identified as correctly targeted at the *hspB1* locus. Clones were microinjected into C57BL/6 blastocysts, and gave rise to male chimeras with a significant ES cell contribution (as determined by an agouti coat colour). Mice were bred to wild-type C57BL/6 mice (*hspB1* ^flox^ mice) and to C57BL/6 mice expressing Cre-recombinase to generate a germline deletion of *hspB1* (*hspB1^+/^* ^del^ mice). Animals were then validated by *Spe* I Southern blot analysis using a 3′ external probe. This confirmed that the founders were heterozygous for the 9.8 kb wild-type fragment with *hspB1* ^flox^ mice displaying a 3.1 kb targeted Neo-excised signal and *hspB1* ^del^ mice showing a 6.5 kb *hspB1*-deleted signal.

*HspB1^+/del^* mice were backcrossed for 12 generations (N = 12) onto a C57BL/6J background (Charles River). Heterozygotes were intercrossed to generate homozygous *hspB1^del/del^* and *hspB1^+/+^* littermate mice, which were expanded by incrossing for use in experiments. All mice were maintained at 21°C±2°C on a 12 h light/dark cycle with feed and water *ad libitum*. All animal experiments were approved by the Kennedy Institute of Rheumatology Ethical Review Process (ERP) Committee and the UK Home Office (PPL 70/7335 and PPL 70/7288).

### Genotyping

Genotyping was performed on tail tip DNA extracted with Wizard SV Genomic DNA purification System (Promega) according to the manufacturer’s instructions. PCR was performed using FailSafe PCR 2x PreMix Buffers (Epicentre Biotechnologies) and the following primers: *HspB1* ^flox^ allele: forward, 5′- TGA CTA GGG GAG GAG TAG AAG GTG GC-3′; reverse, 5′- TCT TGC TAC AAG CCT GGG ACT CTG G-3′; *HspB1* ^del^ allele: forward, 5′-AAT TCT AGC ACC CAC CTG GCA TTC C-3′; reverse, 5′-AGC AGG GAG AGA AAT TAG CAG ATT GGC-3′;. *HspB1* wild-type allele: forward: 5′- CTG GTG CAT CTG AAG GCA GTT ACG G -3′; reverse: 5′- TCT TGC TAC AAG CCT GGG ACT CTG G -3′).

### Isolation and Culture of Primary Murine Embryonic Fibroblasts (MEF)

12.5 day embryos from timed-mated pregnant female mice were isolated and hematopoietic tissue, tubular intestine and central nervous system tissue was removed. The remaining tissue was washed and cells separated in PBS using a syringe (18-gauge needle) and then homogenised in trypsin for 15 min. The suspension was then re-suspended in complete medium and seeded in a 150 mm culture dish (passage zero). At 90% confluence or after a maximum of 5 days cells were trypsinised and filtered through mesh (Passage 1) and maintained in culture or frozen. For routine culture, murine embryonic fibroblasts were passaged every 3 days, seeded at a density of 1×10^6^ cells per 150 mm-dish in DMEM (Lonza) supplemented with 10% (v/v) heat-inactivated FCS (GIBCO), 2 mM L-glutamine, 50 U/ml penicillin and 50 µg/ml streptomycin (complete medium). Unless otherwise stated p3 MEFs were used. For cell proliferation arrest/release experiments MEF were plated at low density (0.5×10^6^ 100 mm-plate), and either synchronised by mitogen deprivation during 72 h (0.1% FCS in DMEM) or by G2/M block in nocodazole (Sigma-Aldrich) for 16 h. Cells were then released by changing the medium for fresh 10% (v/v) FCS-containing DMEM.

### Cell Proliferation Assays

Cell proliferation was assessed by MTT assays and cell counting. MTT assays were performed on cells over the course of 5 days every 24 h. 2000 cells/well (5 wells per condition) were seeded in 96-well plates. Culture medium was replaced every 2 days to avoid mitogen deprivation. At the indicated times the concentration of MTT was added to 0.5 mg/ml (final concentration) and cells incubated for 4 h at 37°C, 5% CO_2_. The formazan salt formed was solubilized by adding HCl and SDS (final concentrations 5 mM and 5% (w/v), respectively) and incubating at 37°C for 16 h. A_570_ was measured using A_690_ as a reference. MEF were also cultured over a period of 9 days and at each passage (every three days), trypsinised, and trypan-blue excluded cells were counted using a haemocytometer. Total numbers of cells were calculated according to the numbers of cells seeded at each passage.

### Fluorescence Labeling Assays

MEF (40 000 cells/well) were seeded on glass coverslips in 12-well plates. For BrdU labeling, cells were incubated with 10 µM BrdU for 2 or 24 h the day after seeding. BrdU incorporation was detected using BrdU Labeling and Detection kit I (Roche). For TUNEL, cells were fixed using 4% (w/v) paraformaldehyde the day after seeding and assayed using ApoAlert DNA fragmentation Assay kit (Clontech). Coverslips were mounted using Prolong Gold antifade reagent (Invitrogen) containing DAPI as nuclear counterstain. Coverslips were examined in triplicate under a fluorescence microscope (Olympus CKX41) and least 500 cells were evaluated per coverslip.

### Western Blotting and Quantitative Reverse Transcription-PCR (qRT-PCR)

For western blotting, cells were lysed with a buffer containing 50 mM Tris-HCl (pH 7.5), 250 mM NaCl, 3 mM EDTA, 3 mM EGTA, 1% (v/v) Triton X-100, 0.5% IGEPAL CA-630, 10% (v/v) glycerol, 1 mM phenylmethylsulfonyl fluoride, 2 µg/ml pepastatin, 10 µM E64, 2 mM NaF, 1 mM Na_3_VO_4_ and 1 µM microcystin. SDS-PAGE and blotting were carried out using standard procedures. Antibodies raised against the following proteins were used: actin (A2103; Sigma-Aldrich), cyclin E (HE12; Cell Signaling), GAPDH (GAPDH-71.1; Sigma-Aldrich), hspB1, (ADI-SPA-801; Enzo), p21^waf1^ (F5; Santa Cruz Biotechnology), p27^kip1^ (C-19; Santa Cruz Biotechnology), poly (ADP-ribose) polymerase (PARP)1 (9542; Cell Signaling), proliferating cell nuclear antigen (PCNA) (PC10; Santa Cruz Biotechnology), and α-tubulin (DM1A; Sigma-Aldrich). RNA was isolated using RNeasy kit (Qiagen) and cDNA was synthesized using the High Capacity cDNA Reverse Transcription Kit (Applied Biosystems). mRNAs were quantified by qRT-PCR using Taqman Gene expression assays and data analysed by relative quantitation (with standard curves) with normalisation to GAPDH mRNA as previously described [34].

### Excisional Cutaneous Wound Healing Model

The dorsal surfaces of anaesthetised female mice 10–12 weeks of age were shaved with clippers and disinfected by swabbing with 70% (v/v) ethanol. Four full thickness excisional wounds (4 mm diameter) were created on the dorsum of the mouse using a biopsy punch. The wounds were digitally imaged on days 0, 3, 5 and 7 post-wounding. Mice were culled on day 1, 3 or 7 and skin sections surgically removed and fixed in 4% (w/v) paraformaldehyde (CellPath) and embedded in paraffin. 5 µm thick sections were cut using an Accu-Cut SRM 200 Rotary Microtome (Sakura). Tissue sections were deparaffinised by heating at 60°C for 30 min before being washed twice in xylene and twice in 95% (v/v) ethanol and stained with Masson’s Trichrome or processed for IHC. The wound areas at d0 to d7 and distances between epithelial tongues in 3 day old wound histological sections were calculated using Image J software (Bethesda, MD). Morphometry was used to measure the areas of partially deposited collagen (weak turquoise staining) which was distinct from fully formed collagen (intense turquoise staining) with ImageJ software. Cytokine expression in wounds was measured by surgically removing tissue from 6 h, d1 or d2 wounds and unwounded tissue distal to the wounds from the same mice as controls and freezing overnight at −20°C in 50 mM Tris HCl, 150 mM NaCl. Tissue was homogenised, transferred to QiaShredder columns (Qiagen) and centrifuged at 13,000 g for 10 min at 4°C. Protein concentrations were determined by Pierce BCA Assay (Bio-Rad) and adjusted to 0.2 µg/ml in 2% (w/v) BSA in PBS sample buffer for ELISA.

### Immunohistochemistry

For heat-induced antigen retrieval slides were immersed in citrate buffer (pH 6.0) and pressure-cooked for 90 s before being allowed to cool to room temperature. HspB1 protein was detected by blocking slides in 10% (v/v) goat serum in PBS for 1 h and incubating with 1∶1000 anti-hspB1 antibody (ADI-SPA-801; Enzo) overnight at 4°C. F4/80 staining was performed by blocking slides as before and incubating with 0.5 µg/ml anti-mouse F4/80 antibody (123101; Biolegend). For elastase detection slides were blocked with 2% BSA and incubated with 0.35 µg/ml anti-neutrophil elastase antibody (68672; Abcam). Endogenous peroxidase was blocked by incubation in 0.3% (v/v) H_2_O_2_ in PBS for 15 min. Slides were incubated with 1∶400 biotinylated goat anti-rabbit IgG (Vector Laboratories) for 1 h and with streptavidin-biotin HRP macromolecular complex (Vector laboratories) for 45 min. All incubations were performed in a humid chamber at room temperature. Slides were rinsed with PBS/Tween-20 (0.05% (v/v)) between incubations. Sections were developed with DAB substrate (Vector laboratories) and counterstained with haematoxylin, cleared in acid alcohol, dehydrated through sequential washes in 50, 70, 95 and 100% (v/v) ethanol and cleared by washing twice in xylene. Coverslips were mounted using a Tissue Tek – Glas G2 (Sakura) and DPX mounting medium (Cell Path). Images were captured using an Olympus BX-51 microscope and Olympus DP-71 camera using the DP Controller software.

### ELISA

Mouse chemokine (C-X-C motif) ligand (CXCL)-1/KC DuoSet (R&D Systems), mouse CXCL-2/MIP-2 DuoSet (R&D Systems), mouse chemokine (C-C motif) ligand (CCL)-2/JE/MCP-1 DuoSet (R&D systems), mouse TNF-α (mono/mono) OptEIA (BD), and mouse IL-6 OptEIA (BD) ELISA kits were used according to the manufacturers’ instructions.

### Air-pouch Model of Acute Inflammation

An air-pouch was created on the dorsal surface of male mice 8–12 weeks of age by injecting 5 ml of sterile air. Four days later the air-pouch was inflated by injecting 3 ml sterile air. The following day 100 µl 1 mg/ml zymosan-A suspension (Sigma-Aldrich) was then injected into the air-pouch. At the desired time-point the mice were culled by CO_2_ asphyxiation. The pouches were injected with 2 ml ice-cold PBS, massaged, and 1 ml of exudate removed. Exudates were centrifuged to pellet the infiltrated cells and supernatants analysed by ELISA. Cells were re-suspended in PBS, stained with trypan blue solution and counted using a haemocytometer.

### Peritoneal Model of Acute Inflammation

Male mice 8–12 weeks of age were injected (IP) with 1 ml of 1 mg/ml zymosan suspension. At the desired time-point the mice were culled by CO_2_ asphyxiation. The peritoneal cavity was lavaged with 5 ml of ice-cold PBS, massaged and the exudate retrieved. Exudates were centrifuged, cells counted as described for the air-pouch model, and cytokine concentrations in supernatants measured by ELISA.

### Statistical Analysis

This was performed by unpaired two-tailed Student’s *t*-test using Prism software (GraphPad). Welch’s correction was used where necessary to correct for unequal variance as determined by F-test using Prism. MTT assay data and IL-6 protein data were analysed using Prism by two-way ANOVA and paired Student’s t-test respectively.

## Results

### Generation of Floxed and *hspb1* ^del/del^ Mice

To examine the function of the small heat shock protein *in vivo hspB1* ^del/del^ mice were generated by introducing *Lox*P sites that flanked the region containing exons 1–3 of the *hspB1* gene (Fig. S1A) and crossing chimeras with CMV-CRE mice. Southern blotting of genomic DNA using *Spe* I restriction digestion and a probe 3′ of the *hspB1* gene (probe Q) showed a 9.8 kb digested fragment for wild-type mice and detection of 6.5 kb and 3 kb fragments confirmed successful generation of heterozygous mice with one wild-type allele and either a deleted or floxed allele respectively (Fig. S1B). Following backcrossing onto a C57BL/6 background, PCR genotyping showed that intercrosses of C57BL/6 *hspB1* ^+/del^ mice gave rise a normal Mendelian ratio of homozygous *hspB1* ^del/del^ animals. The mice were fertile, of normal weight, and those examined up to 18 months of age displayed no obvious abnormalities, or disease symptoms.

### HspB1 Deficiency in Fibroblasts Increases IL-1-induced IL-6 Expression and Inhibits Cell Proliferation

**Figure 1:**
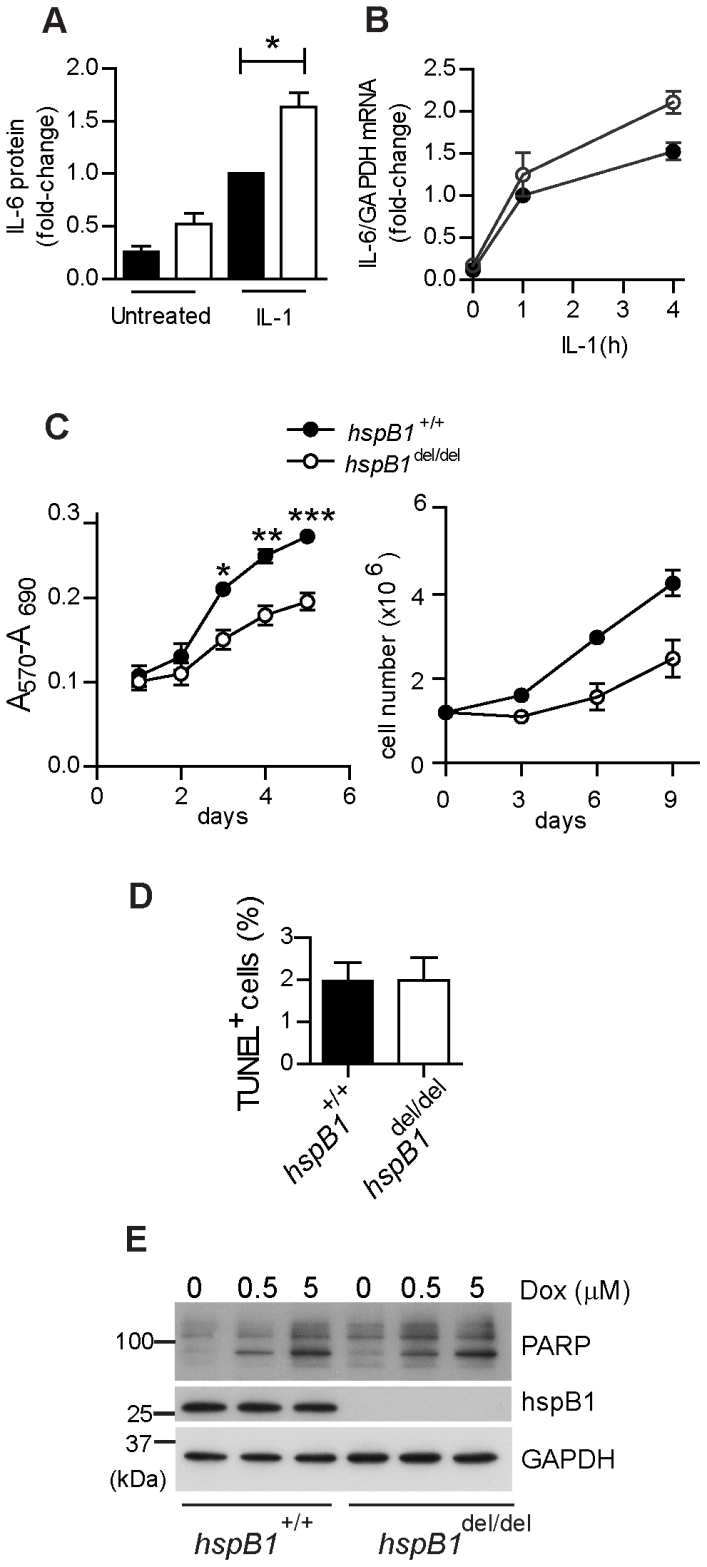
HspB1 deficiency increases IL-1-induced IL-6 expression and inhibits proliferation in fibroblasts. A, Primary wild-type and *hspB1* ^del/del^ MEF were treated with IL-1 (20 ng/ml) for 4 h or left untreated. Graph shows the concentration of IL-6 in culture medium as determined by ELISA and normalised against values for IL-1-treated wild-type MEF for three separate batches of cells (*P<0.05). B, MEF were treated as in (A), lysed, RNA extracted and IL-6 and GAPDH mRNAs quantified by qRT-PCR. Plot shows IL-6 mRNA/GAPDH mRNA normalised to the value for wild-type cells treated with IL-1 for 1 h. C, Growth curve analysis (means±SEM) of wild-type and *hspB1* ^del/del^ MEF determined by MTT assay at different days post-seeding (n = 3; *P<0.05, **P<0.01, ***P<0.001) or by counting trypan-blue excluded cells (n = 2). D, Plot of mean (%) TUNEL-positive (± SEM) cells for three different batches of MEF per genotype. E, MEF were treated with different concentrations of doxorubicin (as indicated) for 8 h to induce apoptosis, or left untreated, cells lysed and lysates analysed by western blot for the full-length and the cleaved form of PARP. Similar results were obtained in three independent experiments.

Given the conflicting reports describing opposing effects of siRNA-mediated depletion on inflammatory gene expression we decided to investigate the effect of complete depletion of hspB1 protein on inflammatory cytokine expression. We were unable to detect the expression of hspB1 protein in murine bone marrow-derived macrophages differentiated with either MCSF, or GM-CSF, or in purified thioglycollate-elicited murine macrophages or neutrophils (data not shown). Therefore to investigate the function of hspB1 protein in inflammatory gene expression primary murine embryonic fibroblasts (MEF) in which the protein was readily detected were used. Inflammatory gene expression induced by IL-1 or TNF in MEF was weak and variable, but there was a reproducible increase in both IL-1-induced IL-6 protein (Fig. 1A) and mRNA (Fig. 1B) expression in hspB1-deficient cells compared to wild-type cells.

**Figure 2:**
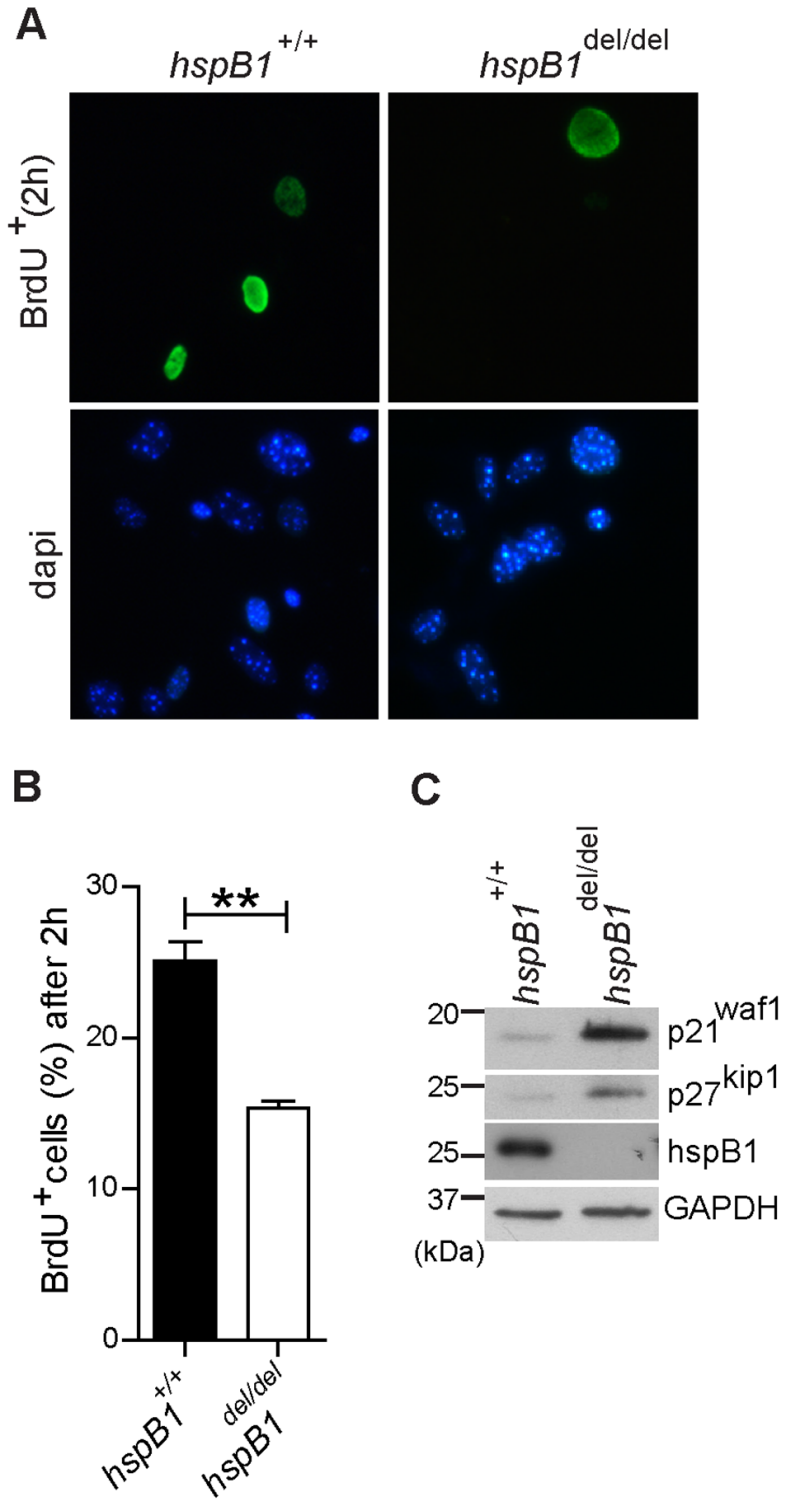
HspB1 deficiency inhibits entry into S phase and increases the expression of p21^waf1^ and p27 ^kip1^. A, BrdU incorporation following a 2-positive cells (mean % ± SEM) from three independent experiments performed; **P<0.01. C, Western blot of asynchronous MEF lysates for p21^waf1^, p27^kip1^, hspB1 and GAPDH as a loading control with molecular weights (kDa) of markers indicated. Western blots are representative of three independent experiments.

During the course of our studies it became apparent that culture dishes of hspB1-deficient MEF appeared to contain fewer cells than those of wild-type MEF. To quantify this primary wild-type and *hspB1* ^del/del^ MEF were seeded at equal densities, allowed to proliferate for 5 days, and cell number was assessed by MTT assay at different times after cell seeding. A_570_–A_690_ was reduced (P<0.001 at d5) for *hspB1* ^del/del^ MEF at d3–5 (Fig. 1C) indicating a difference in live cell numbers or metabolic activity. Counting of trypan-blue-excluded cells over 9 days post-seeding confirmed that *hspB1* ^del/del^ MEF accumulate more slowly over time relative to wild-type MEF (Fig. 1C). Since hspB1 has a well-characterised cytoprotective function it was possible that a greater proportion of *hspB1* ^del/del^ cells were undergoing spontaneous apoptosis than wild-type cells. However, TUNEL assay showed that only approximately 2% of wild-type and *hspB1* ^del/del^ cells were undergoing apoptosis (Fig. 1D). MEF were also treated for 8 h with different concentrations of the DNA-damaging agent doxorubicin, or left untreated, cells lysed, and lysates analysed by western blotting for PARP1 and the caspase-3-cleaved form of the protein that is produced upon induction of apoptosis [35]. In the absence of doxorubicin, cleaved PARP could not be detected but it was induced to a similar degree by doxorubicin in wild-type and *hspB1* ^del/del^ cells (Fig. 1E). Another possibility was that a greater proportion of *hspB1* ^del/del^ MEF were senescent, but staining for SA-β-galactosidase confirmed similar proportions of senescent cells in the two populations at the third, fifth and eighth passages of wild-type relative to hspB1-deficient MEF (data not shown). BrdU pulse for 2 h showed a decrease (P<0.01) in BrdU incorporation and entry into S phase in *hspB1* ^del/del^ cells relative to wild-type (Fig. 2A and 2B). The defect in proliferation of *hspB1* ^del/del^ cells could be attributed, at least in part, to increased expression of the CDK inhibitor p27^kip1^ in hspB1-deficient cells (Fig. 2C). Increased expression of the CDK inhibitor, p21^waf1^, was also observed (Fig. 2C). The expression of mRNAs for p27^kip1^ and p21^waf1^ was unchanged by hspB1 deficiency (data not shown), suggesting that the stability of these proteins, or their translation is regulated by hspB1.

### HspB1 Expression is Regulated by the Cell Cycle

**Figure 3:**
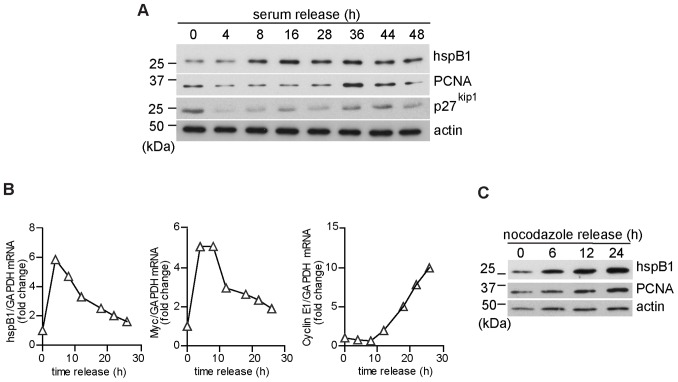
Expression of hspB1 protein and mRNA is controlled by the cell cycle. A, MEF were synchronized by serum starvation for 48% FCS-containing medium and analysed by western blot for hspB1, PCNA, p27^kip1^ and actin. B, Comparison of the expression of mRNAs for hspB1 and the cell cycle-regulated genes, Myc, and Cyclin E1 determined by qRT-PCR and normalized to GAPDH in a representative synchronized MEF serum release time course. C, Western blot for hspB1, PCNA and loading control, actin, in lysates of MEF following release from nocodazole G2/M block (40 ng/ml). All western blots shown are representative of at least two independent experiments.

To explore whether the expression of hspB1 itself is under the control of the cell cycle, MEF were seeded, arrested in G0 by serum starvation for 72 h, and released by re-addition of serum. Western blotting of cell lysates showed that hspB1 expression increased following serum release, peaking at 16 h (Fig. 3A). A reduction in the expression of p27^kip1^ protein following serum release and an increase in PCNA at 36 h confirmed entry into the cell cycle and progression into S phase, respectively (Fig. 3A). HspB1 mRNA was also induced following serum re-addition, peaking at 4–8 h serum release (Fig. 3B), earlier than cyclin E1, but with a similar profile to c-Myc mRNA (Fig. 3B). To examine whether the effects on the expression of hspB1 were specific to growth factors in serum, or due to re-entry of cells into the cell cycle, MEF were arrested in G2/M by nocodazole treatment for 16 h, washed and complete medium replaced to allow cell cycle re-entry. HspB1 protein expression was then analysed by western blot. This showed the induction of hspB1 protein expression following the removal of nocodazole from the cells (Fig. 3C) and confirmed that hspB1 expression is controlled by the cell cycle. The induction of PCNA following nocodazole release confirmed that cells had re-entered the cell cycle (Fig. 3C).

### Delayed Wound Healing in *hspb1* ^del/del^ Mice

**Figure 4:**
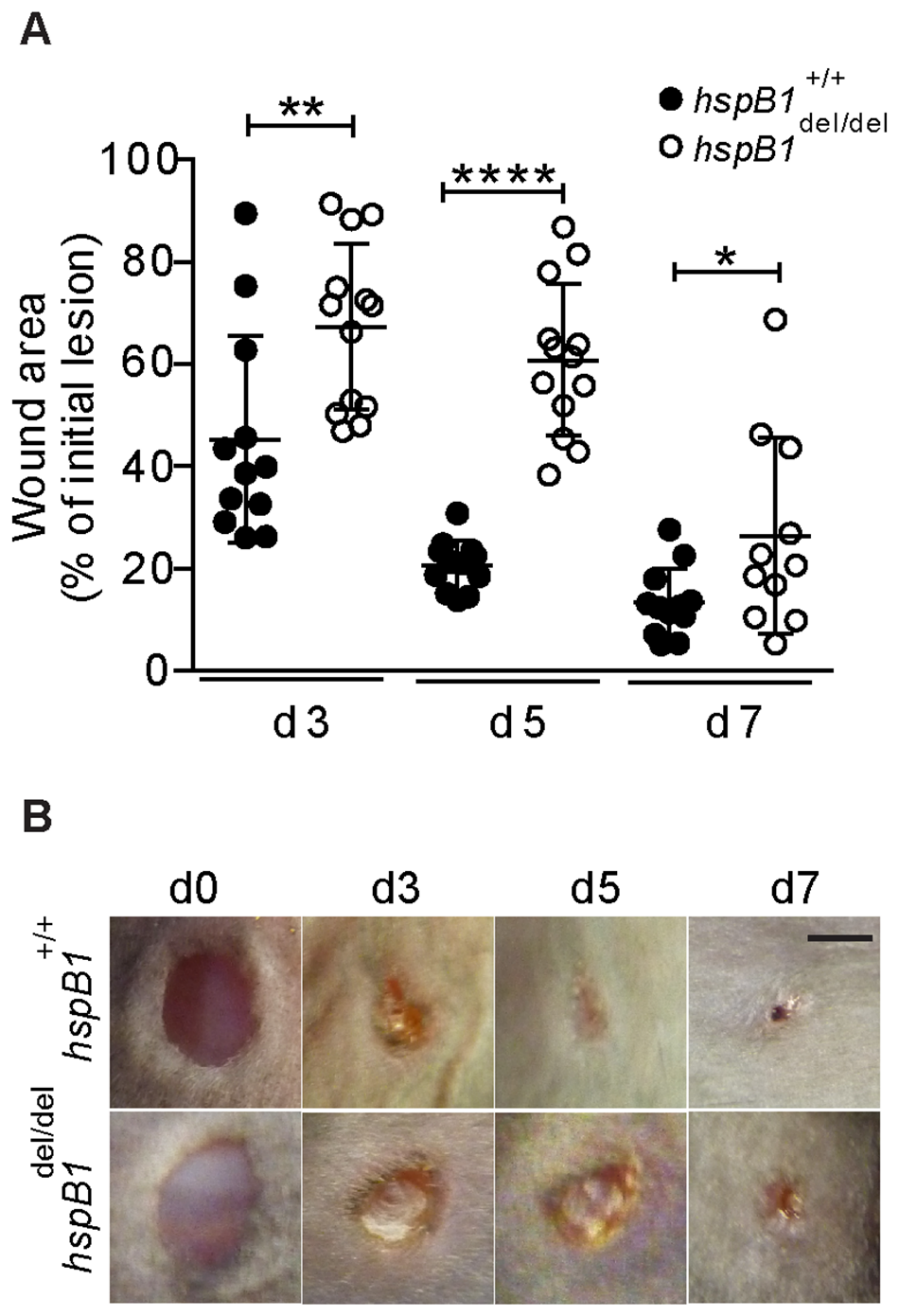
HspB1 deficiency impairs excisional cutaneous wound healing. A, Four 4–14 week-old female wild-type and *hspB1* ^del/del^ mouse. Digital images were taken of each wound at d0, d3, d5 and d7 post-wounding (n = 3–4 mice). Individual wound areas were tracked over time and plotted as a percentage of the intial individual lesion areas±SD (*P<0.05, **P<0.01, ****P<0.0001). B, Digital images of wounds (representative of ≥14 wounds); bar = 2 mm.

The defect observed in cell proliferation prompted us to investigate the function of hspB1 in wound healing. This was investigated by creating wounds on the dorsal surface of wild-type and *hspB1* ^del/del^ mice using a 4 mm biopsy punch. Digital images of wounds were captured over a period of 7 days and wound areas measured and plotted as a percentage of the area of the intial lesion. There was a significant delay in the rate of wound-healing in *hspB1^del/del^* compared with wild-type mice. In the knockout animals wound areas were 1.6-fold larger (P<0.01), 2.9-fold larger (P<0.0001) and 2.2-fold larger (P<0.05) at days 3, 5 and 7 respectively after wounding (Fig. 4A). Digital images of representative wounds over seven days post-injury are shown (Fig. 4B).

### Expression of hspB1 Protein in Unwounded and Wounded Murine Skin

**Figure 5:**
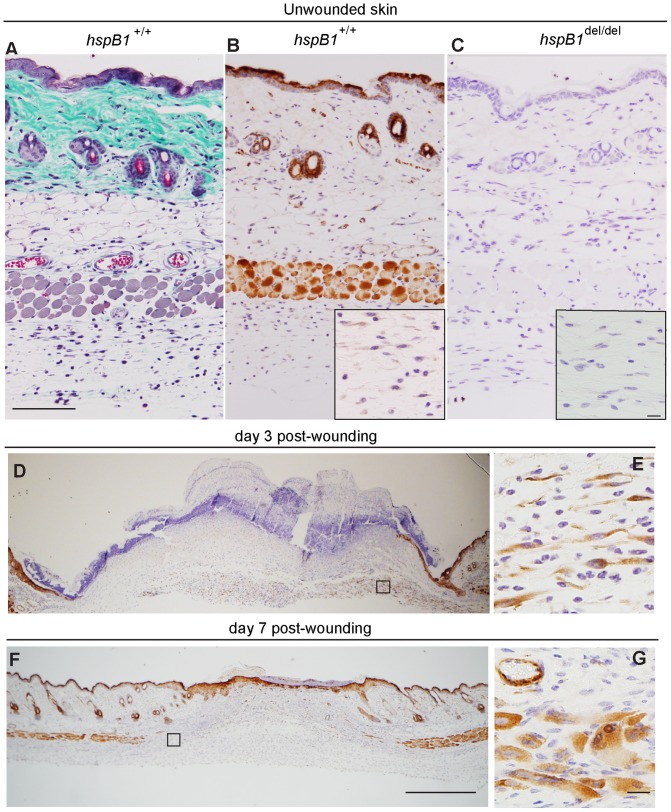
HspB1 protein expression in unwounded and wounded murine skin. A, Unwounded female wild-type skin was stained with Masson’s trichrome; (bar = 100 µm). B, HspB1 was detected by IHC in unwounded female wild-type. Inset: High power image showing hspB1 staining in cells with fibroblast-like morphology in connective tissue beneath panniculus carnosus. C, Unwounded female *hspB1* ^del/del^ skin showing lack of staining with anti-hspB1 antibody. Inset as for (B); (bar = 10 µm). D, HspB1 staining at d3 post-wounding showing expression in epithelial tongues and cells in granulation tissue (representative wounds from 11 mice in three experiments) E, High power image of boxed region indicated in (D) showing hspB1 expressing cells with fibroblast-like morphology in granulation tissue. F, hspB1 staining at d7 showing expression in newly formed muscle, and epithelium (representative wounds from 7–8 mice in two experiments); (bar = 500 µm). G, High power image of boxed region indicated in (F) showing expression of hspB1 in newly formed skeletal muscle and microvasculature; (bar = 10 µm). 11–14 week age-matched female wild-type mice were used.

To determine the expression of hspB1 protein in murine skin wild-type unwounded skin was paraffin-embedded, sectioned, and stained with Masson’s trichrome to clearly delineate the tissue architecture (Fig. 5A) with staining of subsequent sections with an anti-hspB1 antibody (Fig. 5B). This identified expression of hspB1 protein in stratum corneum, epidermis, hair follicles and skeletal muscle as reported previously (Fig. 5A and 5B) [6]. No staining by anti-hspB1 antibody could be detected for *hspB1* ^del/del^ skin when used at the same concentration and the structure of the skin tissue appeared normal (Fig. 5C). HspB1 was also detected in cells with fibroblast-like morphology in connective tissue beneath the panniculus carnosus (Fig. 5B inset) and in capillaries (Fig. 5B), the latter identified by staining of red blood cells inside the vessels with Masson’s trichrome (Fig. 5A).

To determine the pattern of hspB1 expression post-wounding, immunohistochemistry was performed at d1, d3 and d7 post-wounding in wild-type mice. At d1 there was no obvious induction of hspB1 protein expression (data not shown). At d3, hspB1 staining was evident in epithelial tongues (Fig. 5D) and in cells with fibroblast-like morphology (Fig. 5D) shown at high magnification in Fig. 5E. At d7 strong staining was observed in fully formed epithelium (Fig. 5F), expanding skeletal muscle (Fig. 5F and 5G) and in new microvasculature (Fig. 5G). HspB1 was also detected in regions of skin distal to the wounds themselves in stratum corneum, muscle, hair follicles, and in cells lining large capillaries at d3 and d7 (Fig. 5F and data not shown), as in unwounded skin (Fig. 5B). No hspB1 staining was seen in wounds from mutant mice (data not shown), confirming the efficiency of the genetic deletion.

### Wounds from *hspb1* ^del/del^ Mice Display Reduced Collagen Deposition and Re-epithelialisation but Increased Cellular Infiltration

**Figure 6:**
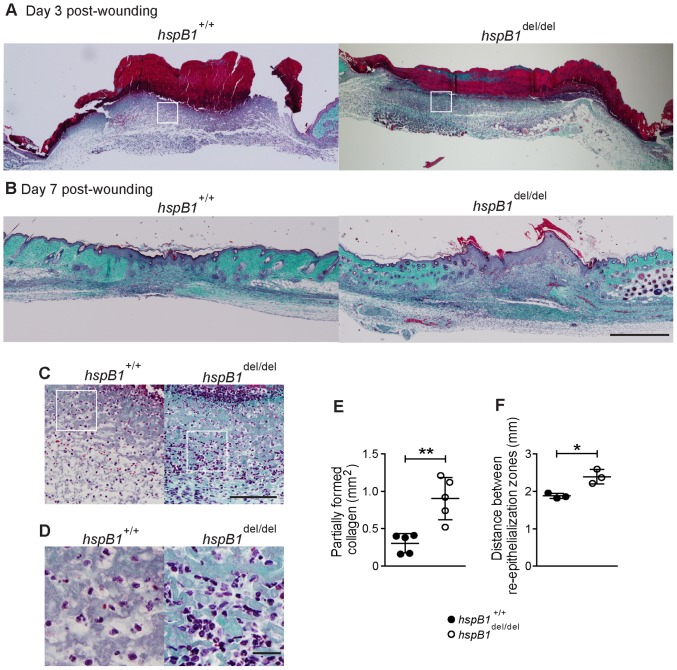
Histological analysis showing reduced re-epithelialisation, impaired collagen deposition and increased cellular infiltration in *hspB1* ^del/del^ relative to wild-type wounds. A, Masson’s trichrome staining of wild-type and *hspB1* ^del/del^ d3 and B, d7 wounds as in Fig. 5; bar = 500 µm. C, High power images of cellular infiltrate from boxed regions indicated in (A) are shown (bar = 100 µm). D, as for (C) but at higher magnification showing cells with multi-lobed nuclei characteristic of neutrophils; (bar = 20 µm).E, Plot showing mean areas of incomplete collagen deposition in d7 wild-type and *hspB1* ^del/del^ wounds (n = 5); **P<0.01. F, Plot of mean distance±SEM (n = 3 mice per group) between re-epithelialisation margins (re-ep) in wild-type and *hspB1* ^del/del^ mice in d3 wounds; (*P<0.05 calculated by Student’s *t*-test of 12 individual wounds per group from 3 experiments). 11–14 week age-matched female wild-type and *hspB1* ^del/del^ mice were used.

To investigate the effect of hspB1 deficiency in wound healing in more detail, sections from wild-type and hspB1-deficient mice were also stained with Masson’s trichrome to identify collagen, epithelium, and capillaries and cellular infiltrate. At d3, Masson’s trichrome staining of wild-type and *hspB1* ^del/del^ skin showed discrete collagen staining at the periphery of the wound, scab formation and proliferating dermis and epithelial tongues (Fig. 6A). Another striking feature of the histology was the increased cellular infiltrate in hspB1-deficient wounds at d3 (Fig. 6A). At high magnification multi-lobed nuclei, characteristic of neutrophils, were observed in infiltrating cells (Fig. 6D). At d7, histological analysis showed that approximately half of wild-type wounds displayed near-complete healing, with the other half displaying incomplete remodelling of the epithelial layer and incomplete collagen deposition. A representative image of a d7 wild-type wound showing partially re-modelled epidermis and advanced collagen distribution is shown (Fig. 6B). Morphometry showed that the area of regions displaying complete collagen deposition were reduced (data not shown) and those displaying only partial collagen deposition were increased (P<0.01) in *hspB1* ^del/del^ wounds (Fig. 6E). None of the d7 *hspB1* ^del/del^ wounds displayed complete healing (Fig. 6B and data not shown). These wounds displayed reduced remodelling of epithelial layers and incomplete collagen deposition (Fig. 6B). The mean distance between epithelial tongues at d3 was greater (P<0.05) in the mutant animals indicating delayed re-epithelialisation (Fig. 6F).

### Neutrophil Infiltration is Increased and Macrophage Infiltration is Slightly Decreased in *hspb1* ^del/del^ Wound Granulation Tissue

**Figure 7:**
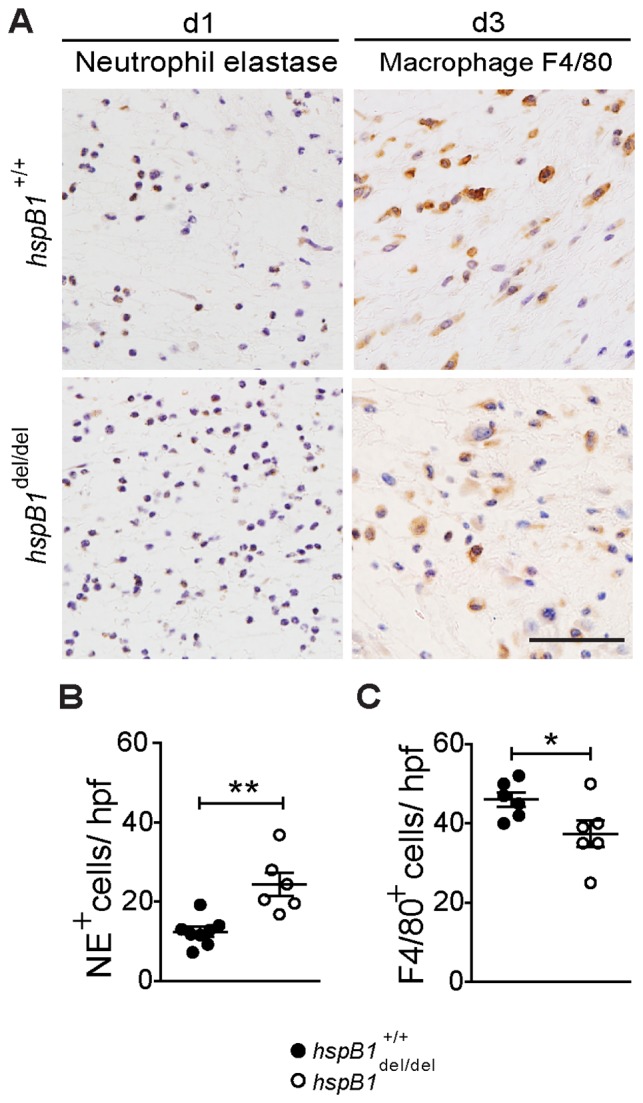
HspB1 deficiency results in increased neutrophil but slightly decreased macrophage infiltration of wounds. A, Neutrophil infiltration in d1 wound granulation tissue assessed by IHC for neutrophil elastase (NE) and macrophage infiltration at d3 detected by F4/80 staining. B, Plot shows elastase positive neutrophils (NE+) per high power field (hpf); 7 fields/wound; 2 wounds per mouse; n = 4 mice; **P<0.01. B, Plot as for (B) showing F4/80-positive cells/hpf; *P<0.05.

To investigate more rigorously whether the infiltration of wounds by neutrophils is affected by hspB1 deficiency, d1 and d3 wound granulation tissue was analysed by immunohistochemistry for neutrophil elastase. Neutrophil infiltration was found to be increased in d1 *hspB1* ^del/del^ wounds relative to wild-type wounds (P<0.01) (Fig. 7A and 7B). It was possible that the effects observed were due to increased numbers of circulating neutrophils rather than an effect on inflammatory responses. However, we confirmed that there was no difference in the numbers of circulating neutrophils in wild-type and *hspB1* ^del/del^ mice (data not shown). At d3, no elastase-positive cells could be detected in granulation tissue of wild-type or hspB1-deficient mice (data not shown).

Macrophage infiltration was also examined in granulation tissue at d1 and d3 post-wounding by F4/80 staining. This showed that there were only low numbers of infiltrating macrophages in d1 granulation tissue (data not shown) but significant infiltration was observed at d3 (Fig. 7A and 7C). There was only a slight decrease (P<0.05) in macrophage numbers in *hspB1* ^del/del^ wounds relative to wild-type wounds (Fig. 7C).

### HspB1 Deficiency Results in Increased Cytokine Expression Early in the Wound Healing Process

**Figure 8:**
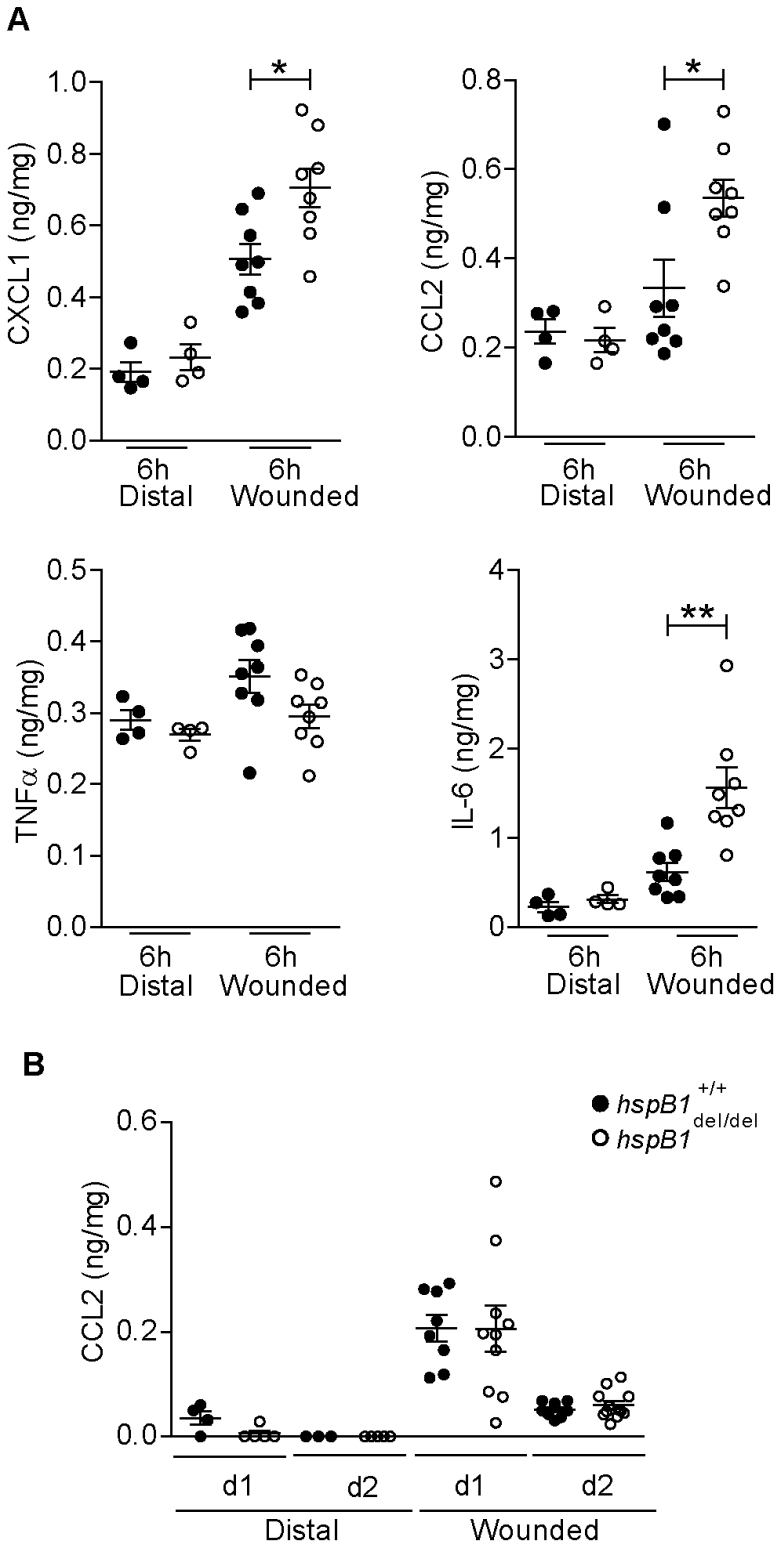
HspB1 deficiency promotes cytokine expression at 6-wounding. A, Plots of cytokine protein expression in homogenised 6(wounded) and distal unwounded tissue (distal) measured by ELISA and expressed as amount of cytokine protein/total protein in wound tissue sample analysed (**P<0.01; *P<0.05). B, Plot of CCL2 protein expression as in (A) but for d1 and d2 post-wounding.

To investigate whether inflammatory cytokine expression is affected by hspB1 deficiency, tissue was harvested 6 h post-wounding, homogenised, and cell lysates analysed by ELISA for CXCL-1, CCL-2, IL-6 and TNF protein. This showed a statistically significant increase in CXCL-1 (P<0.05), CCL-2 (P<0.05), IL-6 (P<0.01) but not TNF protein expression in hspB1-deficient wounds relative to those from wild-type mice (Fig. 8A). While the increase in CXCL1 production in *hspB1* ^del/del^ wounds is consistent with increased neutrophil infiltration the increase in the monocyte chemoattractant, CCL2, was puzzling given that there was a small decrease in the numbers of infiltrating macrophages in *hspB1* ^del/del^ wounds relative to wild-type wounds at d3 post-wounding. Therefore CCL2 and CCL3 expression was also measured at later times more relevant to the peak of macrophage infiltration. CCL2 and CCL3 proteins were induced by wounding at d1 and d2 but no significant effect of hspB1 deficiency on their expression was observed (Fig. 8B and data not shown).

### Effect of hspB1 Deficiency on Cellular Infiltration in the Zymosan Air-pouch Model

**Figure 9:**
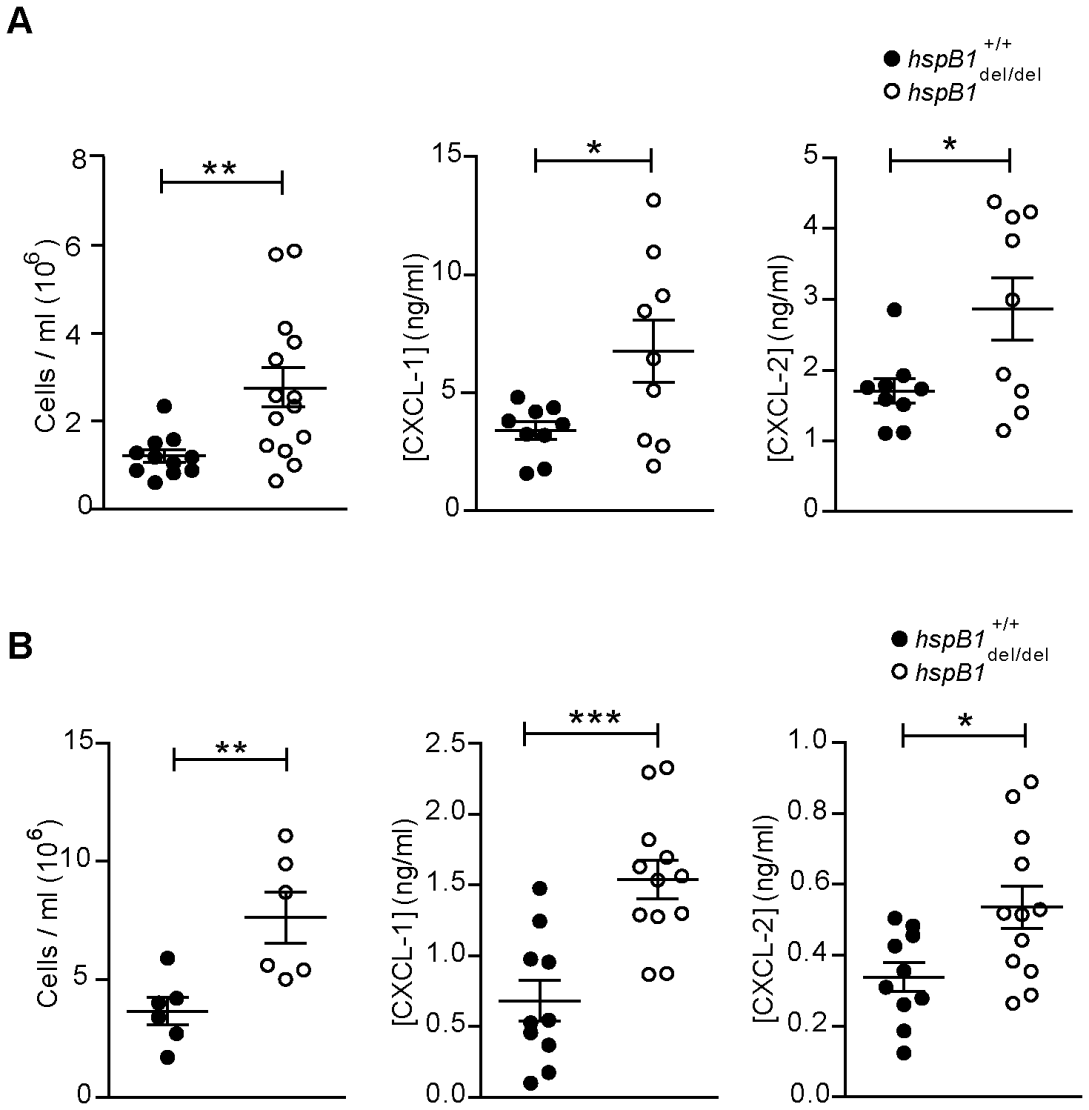
HspB1 deficiency increases neutrophil infiltration and chemokine expression in the zymosan-induced air-pouch and peritonitis models of acute inflammation. A, Air-pouches were created on the dorsal surfaces of 10–12 week old male wild-type (closed circles) *hspB1* ^del/del^ (open circles) mice and were injected with 100 µl of 1 mg/ml zymosan. Exudates were retrieved at the indicated times. Plot of number of trypan blue-negative infiltrating cells counted with a haemocytometer (n = 5–6 mice per group for 2 h post-zymosan; n = 9–14 from two experiments each for all other times). Plots of CXCL-1, and CXCL-2 protein in exudate supernatants measured by ELISA at 1 h post-zymosan (n = 9 from two experiments). Graphs show mean±SEM. B, 1 ml of zymosan (1 mg/ml) was injected into the peritoneal cavities of 10–12 week-old male wild-type and *hspB1* ^del/del^ mice. Plot of number of infiltrating cells with time of zymosan treatment indicated (n = 4–6) and CXCL-1 and CXCL-2 protein in exudates at as for (A) (n = 10–12 from two experiments); *P<0.05, **P<0.01, ***P<0.001.

To test whether the regulation of inflammation in wound healing by hspB1 represents a general mechanism controlling chemokine expression and neutrophil infiltration we investigated zymosan-induced inflammation in the air-pouch model [36]. In this, a cavity is created on the dorsal surface of the mice, and granulation tissue is allowed to form over a period of five days, before challenge with zymosan, a ligand for dectin-1 and TLR 2/6. The current dogma is that zymosan induces the early expression of TNF in resident macrophages; the TNF produced then induces the expression of chemokines by connective tissue cells and primes the vascular endothelium, which together result in the infiltration of neutrophils into the air-pouch cavity [37]. Thus the model provides a robust and reliable method to quantify cellular infiltration and *in vivo* cytokine production in the acute inflammatory response. Air-pouches were created and allowed to form over 3 days before challenge with zymosan. Cells infiltrating the air-pouch at 4 h post-zymosan were counted. A statistically significant increase in cell number at 4 h (2.3-fold; P<0.01) was observed in *hspB1* ^del/del^ mice (Fig. 9A). No significant cellular infiltration was detected in mice injected with PBS vehicle (data not shown). FACS analysis with Ly-6G (Gr-1) staining confirmed that at 4 h post-zymosan neutrophils comprised >90% of the cellular infiltrate in both wild-type and *hspB1* ^del/del^ mice (data not shown), excluding the possibility that the increased cell counts in hspB1-deficient animals are due to infiltration by some other cell type.

The effect of hspB1 deficiency on chemokine expression in the air pouch model was also examined. CXCL-1 and CXCL-2 protein concentrations in exudate supernatants from mice challenged with zymosan for 4 h were measured by ELISA. CXCL-1 and CXCL-2 displayed low levels at 4 h and in vehicle controls (data not shown). Expression of both chemokines was stronger at 1 h post-zymosan in both wild-type and *hspB1* ^del/del^ mice (Fig. 9A). Both peak CXCL-1 and CXCL-2 production was increased by 2.0-fold (P<0.05) and 1.7-fold (P<0.05), respectively, in *hspB1* ^del/del^ mice compared to wild-type controls (Fig. 9A).

### *HspB1* ^del/del^ Mice Display Increased Inflammation in the Zymosan-induced Model of Peritonitis

The inflammatory response to zymosan in the air-pouch model is primed by the prior formation of granulation tissue at the site of air-pouch creation. Thus, given the function of hspB1 in regulating cell proliferation, it was possible that variability in cellular infiltration and cytokine expression in *hspB1* ^del/del^ mice in this model (Fig. 9A) was attributable to differences in formation of granulation tissue. To address this, the peritonitis model of acute inflammation was used which involves direct injection of an inflammatory agent into the peritoneal cavity. As for the air-pouch system, the peritonitis model also allows investigation of the acute inflammatory response and to monitor leukocyte trafficking and cytokine expression. Furthermore, the employment of the same inflammatory stimulus allowed us to relate any results directly to the air-pouch experiments. As for the air-pouch model, intraperitoneal injection of zymosan resulted in a statistically significant 2.1-fold (P<0.01) increase in infiltrating cells at 4 h post-zymosan in *hspB1* ^del/del^ mice compared to wild-type controls (Fig. 9B). As seen in the air-pouch model, *hspB1* ^del/del^ mice displayed similar populations of infiltrating neutrophils at 4 h post-zymosan (data not shown). The production of the chemokines CXCL-1 and CXCL-2 at 1 h post- zymosan was also significantly increased by 2.3-fold (P<0.001) and 1.6-fold (P<0.05) respectively, in *hspB1* ^del/del^ mice compared to wild-type (Fig. 9B).

## Discussion

The function of hspB1 in cell proliferation has remained obscure because many studies have been complicated by the anti-apoptotic function of the small heat shock protein. HspB1 does not appear to exert a cytoprotective function in MEF as TUNEL staining indicated a similar low number of cells undergoing apoptosis in wild-type and *hspB1* ^del/del^ cultures. The lack of a cytoprotective function of hspB1 against the effects of doxorubicin in primary MEF observed here is in agreement with a previous report showing that primary *hspB1* ^−/−^ MEF do not display increased sensitivity to several inducers of apoptosis [6]. The effect of hspB1 deficiency on the cytotoxic effects of doxorubicin in cells with altered p53 status remains to be determined.

The only mechanism described so far for cell cycle control by hspB1 pertains to conditions of cell stress [38]. We found using BrdU incorporation assays that entry into S phase is promoted by hspB1 and that it therefore controls cell proliferation directly. We found that hspB1 deficiency increased the expression of p27^kip1^ under standard tissue culture conditions, as shown previously for siRNA-mediated depletion of hspB1 under conditions of cell stress [38]. We also found that expression of another key CDK inhibitory protein, p21^waf1^, was increased in hspB1-deficient MEF. The lack of regulation of p21^waf1^ mRNA by hspB1 suggests that as reported for the regulation of p27^kip1^ by hspB1 [38] and cyclin D1 by the close relative, αB-crystallin [39], hspB1 may promote the ubiquitination and degradation of p21^waf1^ protein. The expression of p27^kip1^ protein is acutely regulated by the cell cycle, with high expression in G0 and lower levels as cells progress through cell cycle [40], [41]. Overexpression of p27^kip1^ or p21^waf1^ has previously been shown to have a profound effect on proliferation and to cause cell arrest [42]. The timing of hspB1 expression in early G1 of the cell cycle is appropriate for it to promote degradation of CDK inhibitor proteins and entry into S phase. Our results showing that hspB1 directly regulates cell proliferation are in agreement with two recent reports showing that hspB1 silencing by RNAi inhibits tumour growth [20] and a prostate cancer cell proliferation [19].

The function of hspB1 in inflammation was previously unclear as studies using RNA interference to deplete the protein have given inconsistent results [2], [30], [31], [32], [33]. In transformed and tumour-derived cell lines we and others have shown that siRNA-mediated knockdown of hspB1 protein inhibited inflammatory gene expression [2], [20] by diminishing upstream pro-inflammatory signalling [2], [30]. We previously showed that depletion of hspB1 in HeLa cells inhibited the induction by IL-1 of COX-2, IL-6 and IL-8 [2]. In hspB1-depleted HeLa cells, inflammatory mRNAs were destabilised and this was attributed to a reduction in MK2 and p38 MAPK activation arising from inhibition of signalling by TAK1 itself, or that between the MAPK kinase kinase and the IL-1 receptor [2]. Depletion of hspB1 increased cytokine expression in primary human keratinocytes [33] but inhibited it in human dermal fibroblasts [2] and human umbilical vein endothelial cells [31]. We used a complete genetic knockout of the *hspB1* gene in mice to investigate the function of the protein *in vivo* and our results are therefore not compromised by siRNA-mediated off-target effects or artefacts arising from the expression of supra-physiological levels of the protein. We found that in both cultured primary MEF and *in vivo* hspB1 has an anti-inflammatory function and limits inflammatory gene expression. Our results from experiments on the genetically deleted strain remove the uncertainty surrounding the function of hspB1 in inflammation arising from siRNA-mediated depletion experiments.

Activation of the p38 MAPK pathway by pro-inflammatory stimuli [22], [23] and in the G1 phase of the cell cycle [43] results in phosphorylation of the small heat shock protein. Unphosphorylated hspB1 exists in cells as large 24-mer complexes which disaggregate to dimers following phosphorylation [44], [45]. It is possible that phosphorylation increases the bioavailability of hspB1 and thereby increases its ability to suppress inflammatory gene expression and promote cell proliferation.

As might be predicted for a phenotype involving excessive inflammation and a reduced rate of cell proliferation, a statistically significant increased wound area was found at d3, d5 and d7 post-wounding in mice lacking hspB1 relative to wild-type. As seen in air-pouch and peritonitis models, CXCL-1 expression and subsequent neutrophil influx at wound sites were increased in *hspB1* ^del/del^ mice compared to wild-type mice. Neutrophil depletion has previously be shown to accelerate wound healing in mice and it is thought that excessive neutrophil infiltration inhibits the wound healing process [46]. The defect in wound healing arising from hspB1 deficiency could thus be partially explained by increased neutrophil infiltration of wounds. In contrast, macrophage infiltration was only reduced by ∼20% in d3 wounds in *hspB1* ^del/del^ mice and the expression of CCL2 and CCL3 at relevant times post-wounding appeared to be unaffected by hspB1 deficiency. Other effects of hspB1 deficiency may also contribute to the impairment of wound healing in hspB1-deficient mice. Firstly, the defect in proliferation of *hspB1* ^del/del^ cells may be a contributing factor. The reduced rate of proliferation of *hspB1* ^del/del^ cells is entirely consistent with the reduced rate of re-epithelialisation observed in *hspB1* ^del/del^ wounds *in vivo*. Reduced proliferation of *hspB1* ^del/del^ fibroblasts *in vitro* suggests that these cells may proliferate more slowly in hspB1-deficient wound granulation tissue. The induction of hspB1 protein in proliferating cells also correlates well with the induction of hspB1 protein in cells with fibroblast-like morphology at wound sites *in vivo*. Secondly, hspB1 deficiency appears to inhibit the deposition of collagen at d7 post-wounding. It remains to be determined if this is a direct or indirect effect of hspB1 deficiency. Thirdly, it is possible that imbalance in the expression of cytokines such as IL-6 which is increased in the early phase of the inflammatory response in *hspB1* ^del/del^ mice, may further contribute to the defective wound healing phenotype.

In conclusion, our findings demonstrate for the first time that hspB1 has a number of important physiological functions, including suppressing cytokine expression, inhibiting neutrophil infiltration, and promoting cell proliferation which together may contribute to the acceleration of wound healing.

## Supporting Information

Figure S1 Generation of hspB1^del/del^ mice.
(TIF)
