# Supplementary figures and images for: Heat Shock Protein B1-Deficient Mice Display Impaired Wound Healing
Source: PLoS One. 2013 Oct 15;8(10):e77383. doi: 10.1371/journal.pone.0077383 (PMC3797036; doi:10.1371/journal.pone.0077383)

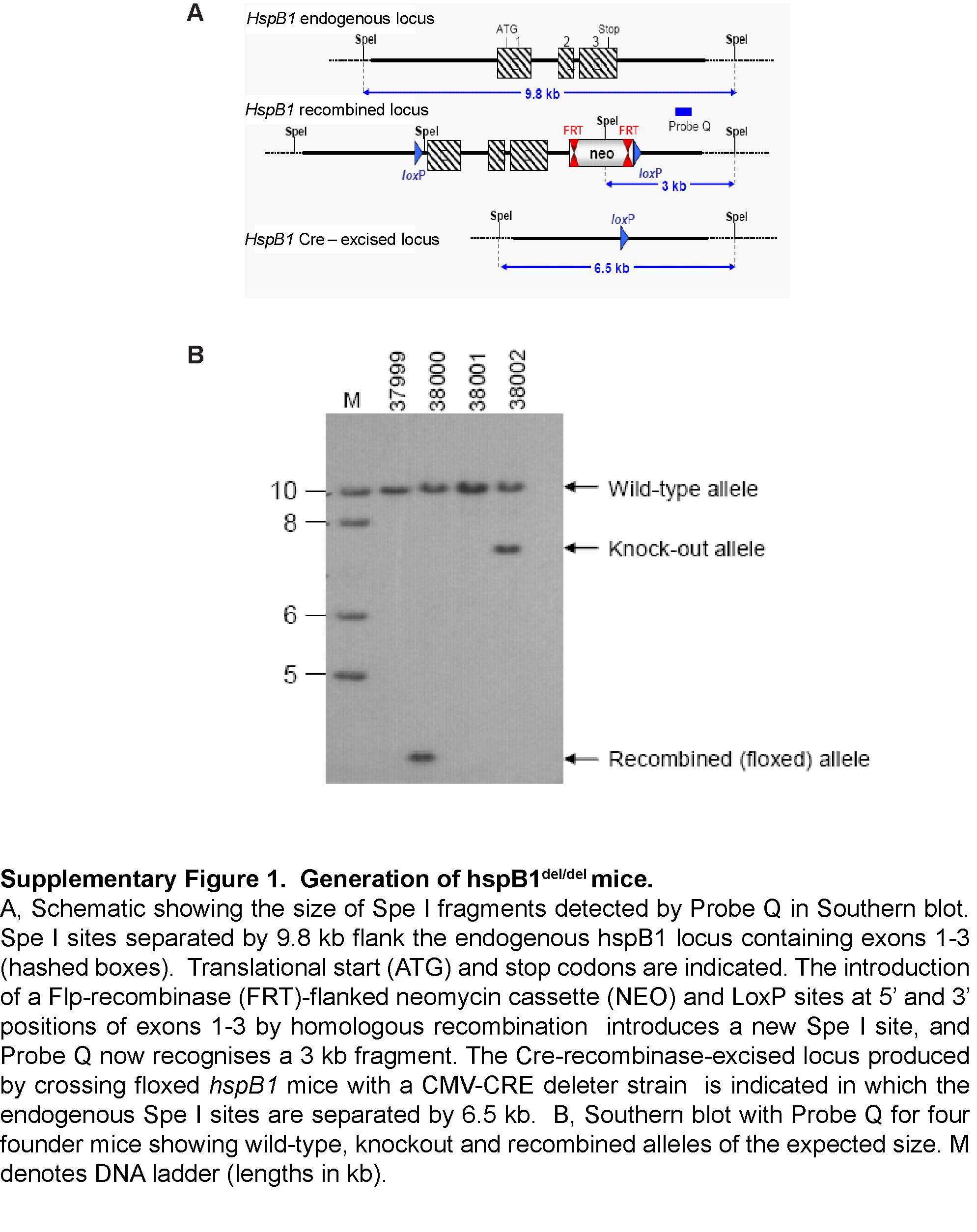

Supplement: Figure S1 — Generation of hspB1del/del mice. (TIF) [file pone.0077383.s001.tif]
